# Cyclic Di-adenosine Monophosphate Regulates Metabolism and Growth in the Oral Commensal *Streptococcus mitis*

**DOI:** 10.3390/microorganisms8091269

**Published:** 2020-08-20

**Authors:** Gro Herredsvela Rørvik, Krystyna Anna Liskiewicz, Fedor Kryuchkov, Ali-Oddin Naemi, Hans-Christian Aasheim, Fernanda C. Petersen, Thomas M. Küntziger, Roger Simm

**Affiliations:** 1 Institute of Oral Biology, University of Oslo, 0316 Oslo, Norway; g.h.rorvik@odont.uio.no (G.H.R.); k.a.liskiewicz@odont.uio.no (K.A.L.); a.o.naemi@odont.uio.no (A.-O.N.); h.c.asheim@odont.uio.no (H.-C.A.); f.c.petersen@odont.uio.no (F.C.P.); t.m.kuntziger@odont.uio.no (T.M.K.); 2 Norwegian Veterinary Institute, Pb 750 Sentrum, 0106 Oslo, Norway; fedor.kryuchkov@vetinst.no

**Keywords:** *Streptococcus mitis*, c-di-AMP, diadenylate cyclase, phosphodiesterase, growth, metabolism

## Abstract

Cyclic di-adenosine monophosphate (c-di-AMP) has emerged as an important bacterial signaling molecule that functions both as an intracellular second messenger in bacterial cells and an extracellular ligand involved in bacteria-host cross-talk. In this study, we identify and characterize proteins involved in controlling the c-di-AMP concentration in the oral commensal and opportunistic pathogen *Streptococcus* *mitis* (*S. mitis*). We identified three known types of c-di-AMP turnover proteins in the genome of *S. mitis* CCUG31611: a CdaA-type diadenylate cyclase as well as GdpP-, and DhhP-type phosphodiesterases. Biochemical analyses of purified proteins demonstrated that CdaA synthesizes c-di-AMP from ATP whereas both phosphodiesterases can utilize c-di-AMP as well as the intermediary metabolite of c-di-AMP hydrolysis 5′-phosphadenylyl-adenosine (pApA) as substrate to generate AMP, albeit at different catalytic efficiency. Using deletion mutants of each of the genes encoding c-di-AMP turnover proteins, we show by high resolution MS/MS that the intracellular concentration of c-di-AMP is increased in deletion mutants of the phosphodiesterases and non-detectable in the *cdaA*-mutant. We also detected pApA in mutants of the DhhP-type phosphodiesterase. Low and high levels of c-di-AMP were associated with longer and shorter chains of *S. mitis*, respectively indicating a role in regulation of cell division. The deletion mutant of the DhhP-type phosphodiesterase displayed slow growth and reduced rate of glucose metabolism.

## 1. Introduction

Cyclic-di-adenosine monophosphate (c-di-AMP) is a bacterial nucleotide messenger molecule that participates in both intracellular and extracellular signaling. Intracellularly, it functions as a second messenger, relaying intracellular and extracellular signals into bacterial cell responses. It regulates a broad range of physiological processes such as bacterial growth and cell size, biofilm formation, virulence, potassium homeostasis, central metabolism, antibiotic susceptibility, maintenance of DNA integrity, and natural transformation [1,2,3,4,5,6,7]. C-di-AMP can also act as an extracellular ligand involved in bacteria-host cross-talk, modulating the host’s immune response. It is recognized by the sensor STING and the pattern recognition receptors DDX41, RECON and ERAdP of the host, leading to the induction of a type I interferon response or activation of NF-κB [8,9,10,11,12]. Bacteria have been shown to modulate the host-response by degrading their extracellular c-di-AMP [13]. Mice models have highlighted the importance of the c-di-AMP signaling network in pathogenesis, where elevated c-di-AMP levels lead to attenuated virulence [3,14,15,16,17].

C-di-AMP is synthesized from ATP by diadenylate cyclases that all contain the DAC (DisA_N) domain responsible for the enzymatic activity [1,8,18,19]. Five classes of diadenylate cyclases: DisA, CdaA, CdaS, CdaM and CdaZ have been identified to date and characterized to different extent [1,8,20,21,22,23]. Although they share the DAC domain, they differ in structure, transcriptional and enzymatic regulation as well as distribution among bacterial species [20]. The enzymes that degrade c-di-AMP are referred to as phosphodiesterases, and so far, five classes have been identified: GdpP, DhhP, PghP, CdnP, and AtaC [13,15,16,24,25]. GdpP and DhhP differ in overall protein structure and predicted cellular localization, but both contain the DHH/DHHA1 domain that harbors the enzymatic activity. The enzymatic activity of PghP, CdnP, and AtaC is located in the HD-domain, Metallophos-domain and Phosphodiest-domain, respectively.

The c-di-AMP signaling system has been studied in several gram-positive pathogens including, but not limited to, *Staphylococcus aureus, Listeria monocytogenes, Streptococcus pyogenes* and *Streptococcus pneumoniae* as well as the important pathogen *Mycobacterium tuberculosis* [4,8,15,18,19]. *Streptococcus mitis* (*S. mitis*) is one of the predominant members of the oral microbiota of healthy individuals [26]. It colonizes most surfaces of the oral cavity [26], and a commensal relationship is established between *S. mitis* and the host early in life [27]. However, *S. mitis* is an opportunistic pathogen that can invade the circulatory system and is commonly associated with gram-positive bacteremia in immune compromised individuals and in infective endocarditis [28,29,30]. *S. mitis* belongs to the Mitis group of bacteria and is phylogenetically closely related to *S. pneumoniae*, an important cause of lethal infections in humans [31,32]. In most streptococci, including *S. pneumoniae*, c-di-AMP is produced by a CdaA-type diadenylate cyclase and is broken down to the intermediate product 5′-phosphadenylyl-adenosine (pApA) by Pde1 and further to AMP by Pde2 [15]. In *S. pneumoniae*, Pde2 also degrades c-di-AMP directly to AMP [15].

Since c-di-AMP has been shown to control growth, stress tolerance and virulence in many streptococci, we hypothesize that c-di-AMP is involved in regulating the commensal lifestyle of *S. mitis*. In this study, we identify and characterize the components of the c-di-AMP signalling system of *S. mitis* and determine their role in physiology and growth.

## 2. Materials and Methods

### 2.1. Bacterial Strains and Growth

All strains and mutants used in this study are listed in Table 1. *S. mitis* CCUG 31611 is referred to as the wild type (WT) throughout the text. Streptococci were grown in tryptone soya broth (TSB) (Oxoid, Basingstoke, UK) at 37 °C in a humidified atmosphere of 5% CO_2_, unless otherwise stated. Pre-cultures were prepared by inoculating bacteria from frozen stocks (−80 °C) onto blood agar plates (Blood agar base No.2 (Merck, Kenilworth, NJ, USA) +5% defibrinated sheep blood (Oxoid)), followed by overnight incubation at 37 °C in a humidified 5% CO_2_ atmosphere. A few colonies were inoculated into TSB and grown in liquid cultures to an optical density (OD_600_) ≈ 0.5, before glycerol (15%) was added and the bacterial suspension was aliquoted into Eppendorf tubes. Pre-cultures were stored at −80 °C. For experiments, pre-cultures were diluted ten-fold in TSB and grown to relevant OD_600_. Two *E. coli* strains, DH5α and BL21 (DE3), were used for cloning of genes of interest and expression of recombinant proteins, respectively. Both strains were routinely grown in Lysogeny Broth (LB) containing tryptone (10 g/L; Oxoid), Bacto^™^ Yeast Extract (5 g/L; Becton, Dickinson and Company, Franklin Lakes, NJ, USA), NaCl (10 g/L; Merck), or on LB agar containing bacteriological agar (15 g/L; VWR, Radnor, PA, USA). When required, plasmid maintenance was achieved by supplementing growth media with kanamycin (50 mg/L; MilliporeSigma, Burlington, MA, USA).

### 2.2. Bioinformatics Analyses

BLAST searches were performed to identify putative c-di-AMP turnover proteins in *S. mitis*. The Blossum-62 matrix was used and the E-value threshold was set to 0.001. Since all classes of diadenylate cyclases known to date contain the conserved Pfam:DisA_N domain (PF02457), the amino acid sequence of the DisA_N-domain of SPD_1392 from *S. pneumoniae* D39 was used as query in the BLAST searches to identify putative diadenylate cyclases. To identify putative phosphodiesterases, a representative of each of the GdpP- (SPD_2032), DhhP- (SPD_1153), PgpH- (Lmo1466), CdnP- (Gbs1929), and the AtaC- (Vnz_27310) type phosphodiesterases as well as the DHH/DHH1- (PF01368/PF02272), HD- (PF01966), Metallophos- (PF00149), and Phosphodiest- (PF01663) domains were used as query. Prediction of the domain architecture of putative diadenylate cyclases and phosphodiesterases was performed using the Simple Modular Architecture Research Tool (SMART) [34].

### 2.3. Cloning and Heterologous Expression of cdaA, pde1 and pde2

The open reading frames (ORFs) of *cdaA*, *pde1* and *pde2* as well as the fragments encoding amino acids 94–285 and 103–285 of CdaA and amino acids 53–657 of Pde1 were amplified from genomic DNA of *S. mitis* CCUG 31611 using primers listed in Table S1. Amplicons were cloned into the pET28a(+)KpnI-mut protein expression vector between the KpnI and HindIII sites, using FastDigest^™^ enzymes (ThermoFisher Scientific, Waltham, MA, USA) and the Rapid DNA Ligation kit (ThermoFisher Scientific). The pET28(+)KpnI-mut vector was constructed to simplify sub-cloning of the relevant genes between vectors. Constructs were verified by Sanger sequencing at Eurofins Genomics (Ebersberg, Germany). *E. coli* BL21 (DE3) cells harbouring constructs were grown in LB with 50 mg/L kanamycin at 37 °C and 190 RPM until an OD_600_ of 0.4–0.5. Protein expression was induced by addition of isopropyl β-D-1-thiogalactopyranoside (IPTG; MilliporeSigma) at a final concentration of 50 µM. Protein expression was maintained for 3 h at 37 °C, 190 RPM.

### 2.4. Protein Purification

Bacterial cells were collected by centrifugation at 4000× *g* at 4 °C for 10 min and re-suspended in Equilibration Buffer (1× phosphate buffered saline (PBS; MilliporeSigma), 10 mM imidazole (MilliporeSigma), pH 7.4). The cell suspension was supplemented with (SIGMAFAST^™^ Protease Inhibitor Cocktail Tablets, EDTA-free; MilliporeSigma), lysozyme (0.1 mg/mL; MilliporeSigma), DNaseI (10 µg/mL; Roche, Basel, Switzerland), MgCl_2_ (10 mM; MilliporeSigma), MnCl_2_ (10 mM; Alfa-Aesar, Haverhill, MA, USA). Bacteria were lysed by sonication on ice with 10 pulses, 30 s each with 10 s breaks (Hielscher Ultrasound Technology, Teltow, Germany) and centrifuged at 4000× *g* at 4 °C for 10 min to remove cell debris. Supernatant was filtered through a 0.2 µm Filter Unit (Whatman, Maidstone, UK) before protein purification with 2 mL of HisPur^™^ Ni-NTA Resin (Thermo Fisher Scientific) according to the manufacturer’s instructions. The proteins of interest were eluted with Elution Buffer consisting of PBS (pH 7.4) and 250 mM imidazole. The eluted protein fractions were concentrated by centrifugation at 5000× *g* for 90 min in an Amicon^®^ Ultra Centrifugal Filter Unit with a molecular weight cut-off of 3 kilodaltons (kDa).

Proteins were subjected to an additional purification step through the Superose^™^ 6 Increase 10/300 GL column (GE Healthcare, Chicago, IL, USA) connected to an ÄKTA Pure 25 system (GE Healthcare). The size exclusion chromatography (SEC) buffer consisted of 1× PBS (pH 7.4) with 10% glycerol (Sigma-Aldrich). The purification protocol was run at 0.5 mL/min with isocratic elution through 1.5 column volumes (CV). Proteins were detected at 280 nm wavelength. Peak fractions were collected and protein purity was analysed by SDS-PAGE and Coomassie staining. The identity of the purified proteins was confirmed by western blotting using a mouse anti-His-tag antibody and a secondary HRP-conjugated anti-mouse antibody (Thermo Fisher Scientific). Peak fractions were concentrated with the Amicon^®^ Ultra Centrifugal Filter Unit. Protein concentrations were determined with NanoDrop 2000c Spectrophotometer (Thermo Scientific) and confirmed using the DC Protein Assay (Bio-Rad, Hercules, CA, USA). Protein aliquots were stored at −80 °C until use.

### 2.5. Determination of the Quaternary Protein Structure

The molecular weights of the proteins of interest were determined in comparison to the protein standard, Gel Filtration Markers Kit for Protein (Molecular Weights 12,000–200,000 Da; Sigma-Aldrich). The relevant proteins and the protein standards were separated on a Superose^™^ 6 Increase 10/300 GL column (GE Healthcare) connected to the ÄKTA Pure 25 system (GE Healthcare) using 1.5 CVs of SEC buffer. The void volume (*v*_0_) of the column and the elution volume (*v*_E_) of each protein were determined, and the ratio (*v*_E_/*v*_0_) calculated. A standard curve was derived by plotting the *v*_E_/*v*_0_ against the MW of the reference proteins and used to estimate the molecular weight of CdaA_103–285_, Pde1_53–657_ and Pde2 in solution.

### 2.6. In Vitro Analysis of Enzyme Activity and Enzyme Kinetics Activity Assays

Determination of the enzymatic activities was based on the previously described assays by Bai. et al. [15] with some modifications. The diadenylate cyclase assay (50 µL) for CdaA was performed in Tris-HCl (40 mM; pH 7.5), 100 mM NaCl, *x*Cl_2_ (*x* = Mn, Mg, Co; 10 mM) and ATP (2 mM). The assay buffer was pre-heated to 37 °C prior to addition of CdaA_103–285_ (10 µM). The diadenylate cyclase assay was performed for 24 h at 37 °C with shaking at 300 RPM. Reactions were stopped by heating in a 99 °C water-bath for 10 min. Precipitated proteins were removed by centrifugation at 12,000× *g* for 2 min and 45 µL of the supernatant was used for analysis.

To determine the enzymatic activity of Pde1_53–657_ the reaction mix (10 µL) contained 50 mM Tris-HCl (pH 7.5), 1 mM MnCl_2_ and 0.5 mM c-di-AMP (Biolog, Hayward, CA, USA). The reaction was initiated by addition of 2.5 µM Pde1_53–657_ into the pre-warmed mix, and carried out at 37 °C with shaking at 300 RPM for 30 min.

For Pde2 activity the reaction mix consisted of 50 mM Tris-HCl (pH 8.5), MnCl_2_ (1 mM) and c-di-AMP (0.5 mM). The reaction was initiated by addition of Pde2 (10 µM) into the pre-warmed mix, and incubated at 37 °C with shaking at 300 RPM for 4 h. All samples were inactivated as described above and 8 µL of the supernatants were used for analysis.

Additionally, 2.5 µM Pde1_53–657_ and 10 µM Pde2 were incubated with 0.5 mM pApA (Biolog) for 10 min, with the same buffer composition as mentioned above.

### 2.7. Kinetics Assays

The kinetics assays were performed in a similar way as described for the activity assays with minor changes. CdaA_103–285_ (10 µM) was incubated in MnCl_2_-containing buffer for 2 min with ATP concentrations from 100 to 2000 µM. The reaction volume for CdaA_103–285_ was 25 µL and 22.5 µL supernatant was used for analysis. Pde1_53–657_ (2.5 µM) was incubated with 25–300 μM c-di-AMP for 30 s, while Pde2 (10 µM) was incubated for 10 min with c-di-AMP (50–2000 µM). The kinetics assays with Pde1_53–657_ (5.0 µM) and Pde2 (0.05 µM) together with pApA as substrate were incubated for 5 s for both enzymes. Pde1_53–657_ and Pde2 reactions included 50–500 µM and 10–2000 µM pApA, respectively. Enzyme kinetics reactions with Pde1_53–657_ and Pde2 were inactivated by adding 50 µL of Buffer A pre-warmed to 99 °C. Forty-eight μL of supernatant was used for analysis. The kinetics results were obtained by fitting the data to the Michaelis-Menten equation using Prism 8 (GraphPad Software, San Diego, CA, USA).

### 2.8. Quantification of Nucleotides by Reversed Phase Chromatography

Samples were diluted in Buffer A (KH_2_PO_4_ (150 mM) and KCl (150 mM); pH 7.0) to 100 µl final volume and loaded onto ÄKTA Pure 25 equipped with a UV detector (280 nm) and an ODS Hypersil^™^ 100 mm × 3 mm column (particle size 3 µm; Thermo Fisher Scientific). The Hypersil column was equilibrated with Buffer A before sample application. Nucleotides were eluted with a multistep gradient of Buffer B (85% Buffer A and 15% *v*/*v* acetonitrile; Sigma-Aldrich) according to the following scheme: 0–3% B in 1 CV, 3–9% B in 2 CV, 9–60% B in 3 CV, 60–100% B in 0.5 CV and an isocratic step at 100% B for 1 CV. Nucleotide standards (AMP, ATP, pApA and c-di-AMP) were used as references. Serial dilutions of 10 to 0.078 nmol of each nucleotide were used to generate standard curves.

### 2.9. Mutant Construction

In-frame marker-less deletion mutants of the genes coding for the putative diadenylate cyclase CdaA (SM12261_1351), and phosphodiesterases Pde1 (SM12261_1779) and Pde2 (SM12261_1122) were constructed in *S. mitis* CCUG 31611, as described by Salvadori et al. [33] with minor modifications. Briefly, PCR using the Phusion High-Fidelity DNA-polymerase (Thermo Fisher Scientific) was used to amplify approximately 3000 bp of the upstream and downstream regions flanking the gene of interest using genomic DNA from *S. mitis* CCUG 31611 as template. Primers were designed to generate an overlap between the two fragments, and the fragments were sown together in a second PCR. This resulted in an approximately 6000 bp long PCR-product containing the flanking regions but lacking the gene of interest. Pre-cultures were 100-fold diluted in C+Y_YB_ medium [35] and grown to an OD_600_ nm of 0.04 at 37 °C in a 5% CO_2_ atmosphere. At this point, *S mitis* CCUG 31611 competence stimulating peptide (CSP; EIRQTHNIFFNFFKRR; 300 nM) and the gene deletion amplicon (200 ng/mL) were added, and the incubation was continued for 3 h. Cultures were diluted in PBS, plated on blood agar plates and incubated overnight. Direct colony PCR was performed to identify colonies positive for gene deletion. Positive colonies were re-streaked on blood agar and subsequently stored in glycerol stocks at −80 °C.

The knock-back strains were created as described for the deletion mutants, with the following modifications. A region of DNA consisting of the gene of interest and 3000 bp upstream and downstream of the gene was amplified by PCR using genomic DNA from S. mitis CCUG 31611 and the relevant KO-R-rev and KO-L-for primers (Table S1). The resulting DNA fragment (200 ng/mL) was used in place of the gene deletion amplicon in the transformation procedure as described above. The relevant deletion mutants were used as recipients.

### 2.10. RNA Isolation and Real Time Quantitative PCR (RT- qPCR)

Pre-cultures were inoculated in TSB (1:10) and grown to an OD_600_ of 0.5. Total RNA was isolated with the High Pure RNA-Isolation Kit (Roche), according to the manufacturer’s instructions for bacterial RNA isolation, with an additional mechanical lysis step. The cell pellet was resuspended in 600 µL Tris-HCl pH 8, transferred to Lysing Matrix B tubes (MP Biomedicals™, Irvine, CA, USA) and homogenised at 5000 rpm 2 × 22 sec using a FastPrep-24 homogeniser (MP Biomedicals™). The RNA was quantified and the quality was assessed (Nanodrop 2000C) before cDNA was generated from total RNA using the First Strand cDNA Synthesis Kit as described by the manufacturer (Thermo Scientific). Quantitative Real Time PCR (RT-qPCR) was performed using the PowerUP SYBR Green Master Mix. DNA gyrase A was used as reference gene. The fold difference in transcription between mutants and WT was calculated using the ΔΔCq method.

### 2.11. Colony Size

WT and mutants were inoculated onto blood agar plates and incubated for 24 h. In order to compare the colonies grown under identical conditions, all strains were inoculated onto the same plate. The plates were illuminated from above and below and photographed using a Sony a7r4 camera.

### 2.12. Growth Assays

Pre-cultures were diluted ten-fold in TSB and grown to an OD_600_ of approximately 0.5. Cultures were diluted to OD_600_ of 0.01 and 100 µl of the diluted cultures were transferred to wells of a 96-well microtiter plate. Growth assays were performed at 37 °C in ambient atmosphere in a plate reader (Cytation 3 imaging reader, Biotek, Winooski, VT, USA) for 20 h; measuring OD_600_ every 15 min. Experiments were carried out with shaking for 15 s before each measurement.

### 2.13. Glucose Metabolism

Glucose break-down products were determined as described by Assev et al. [36], with some modifications. Briefly, pre-cultures were diluted ten-fold in TSB and grown to an OD_600_ of 0.45–0.50. Cells were collected by centrifugation at 6000× *g* at 25 °C for 5 min before discarding the supernatant. Cells were washed once in PBS and once in the appropriate assay buffer; Buffer I) PBS supplemented with MgCl_2_ (2 mM), Buffer II) PBS with MgCl_2_ (2 mM) and KCl (2 mM) or Buffer III) PBS with MgCl_2_ (2 mM) and KCl (10 mM). Bacteria were finally resuspended in assay buffer I, II or III and the OD_600_ was adjusted to 2.0. The assay was started by addition of ^14^C-glucose (1.5 mM) and glucose (1.5 mM). PBS with the same concentrations of ^14^C- glucose and glucose was used as a control. Samples were incubated for 60 min at 37 °C. The assay was stopped by addition of ice-cold NaF (20 mM) and the samples were cooled on ice. Bacteria were pelleted at 12,000× *g* at 4 °C for 1 min, and the supernatant was collected and filtered through a 0.45 µm filter. The flow through was stored at −20 °C until further analyses, in which the metabolites were separated by high performance liquid chromatography (HPLC) using a LC-10AT system (Shimadzu, Kyoto, Japan) and an Aminex HPX-87H (300 mm × 7.8 mm) ion exclusion column (mobile phase 1.25 mM H_2_SO_4_, flow rate 0.6 mL/min, 60 °C) and monitored by a radioactivity detector (Ramona Star, Elysia-Raytest, Angleur, Belgium).

### 2.14. C-di-AMP and pApA Profiling by HRMS/MS

Bacteria from pre-cultures were ten-fold diluted in TSB medium and grown to mid-exponential phase (approximately OD_600_ 0.5). Bacteria were centrifuged at 13,000× *g* for 5 min, and resuspended in an extraction buffer consisting of 40% acetonitrile, 40% methanol, and 20% water and metabolites were extracted as described by Burhenne and Kaever [37]. Extracts were evaporated in nitrogen flow at 70 °C and resuspended in 100 ul of water. Insoluble impurities were removed by centrifugation at 15,000× *g* for 5 min, and the supernatants were transferred into HPLC vials.

Targeted analysis was achieved on a Vanquish Horizon UHPLC instrument connected to a Q-Exactive mass spectrometer, equipped with a heated electrospray interface (HESI-II, Thermo Fisher Scientific). Chromatographic separation was conducted using Kinetex F5 column (1.7 µm, 2.1 × 100 mm, Phenomenex, Torrance, CA, USA) at 20 °C and 300 mL/min flow rate. Mobile phase A consisted of aqueous ammonium acetate (10 mM) supplemented by 0.1% of acetic acid. Mobile phase B consisted of pure methanol. Metabolites were separated in a 12 min program with the following UHPLC gradient conditions: isocratic elution using linear gradient from 0 to 8% B over 8 min, followed by a washing step with 80% B for 2 min and equilibrating at 0% B for the next 2 min. Injection volumes were set to 3 µL. The following parameters for the HESI-II source were used: electrospray voltage of 3.0 kV; capillary temperature of 290 °C; S-lens voltage of 60 V; auxiliary gas heater temperature of 350 °C. Positive ion mass spectra were recorded in the parallel reaction monitoring (PRM) mode using the following acquisition parameters: a mass resolution set to 35,000 (at 200 *m*/*z*), an automatic gain control target of 2 × 10^5^ ions, a maximum ion inject time of 128 ms, an isolation width of 0.4 *m*/*z*. The inclusion list included protonated c-di-AMP (659.1) and pApA (677.1) molecules. c-di-AMP ions were fragmented using a normalised collision energy (NCE) of 16 eV and 36 eV, while pApA was fragmented employing a NCE of 16% and 36%. Extracted ion chromatograms (XIC, ±5 ppm) were constructed for the following MS transitions: *m*/*z* 659.1 to *m/z* 330.0598 at NCE of 16%, *m*/*z* 677.1 to *m*/*z* 428.0367 at NCE of 24%, *m*/*z* 659.1 to *m*/*z* 312.0492 at NCE of 36%, and *m*/*z* 677.1 to *m*/*z* 542.0684 at NCE of 16% using Xcalibur v 4.2 (Thermo Fisher Scientific). The first two MS transitions were used for peak area measurements, and the other two were used for the purpose of confirmation. All solvents were LCMS-grade, ammonium acetate and acetic acid, p. a.-grade were bought from MilliporeSigma.

### 2.15. Determination of Chain Length

Pre-cultures were diluted ten-fold in TSB and grown to an OD_600_ of 0.5. One µL inoculation loops were used to spread the cultures on an object glass. The samples were air dried before fixation by passing the object glass through a flame. Bacteria were stained for 60 s by crystal violet, and excess stain was rinsed off with water. Slides were inspected by light microscopy (Nikon), imaged and the chain lengths in each sample were determined by manual counting of the constituting bacteria.

### 2.16. Flow Cytometry Analysis

Flow cytometry analysis was performed to investigate size and chain length of WT and mutants. Bacteria were diluted ten-fold in TSB medium and grown to an OD_600_ of 0.5. Bacteria in medium and medium alone were analyzed with a FACSort flow cytometer (BD Biosciences). Forward and side scatter settings were set, based on analysis of medium alone, to ensure analysis of bacteria only. Forward scatter correlates with bacteria size and side scatter reflect granularity. A total of 100,000 events were analyzed using medium speed. Flow data collection was carried out using the program CellQuest 3.3 (BD Biosciences) and the data generated were further analyzed with the program Kaluza analysis 2.1 (Beckman Coulter, Indianapolis, IN, USA).

### 2.17. Transformation Efficiency

To investigate the ability of the WT and mutants to take up and incorporate foreign DNA into their genomes, transformation efficiency was determined using the transformation protocol described for the construction of mutants. For this experiment, bacteria were transformed using the 5957 bp erythromycin marker amplified from *S. mitis* strain MI 009. The transformation mixtures were serially ten-fold diluted in PBS and plated on blood agar plates with and without erythromycin (10 mg/L). Plates were incubated for 24 h, the number of colony forming units (CFU) was determined and the ratio of CFU on agar with and without erythromycin was calculated. This measurement of transformation efficiency was compared for the mutants and WT.

## 3. Results

### 3.1. Identification of c-di-AMP Turnover Proteins in S. mitis

**Figure 1:**
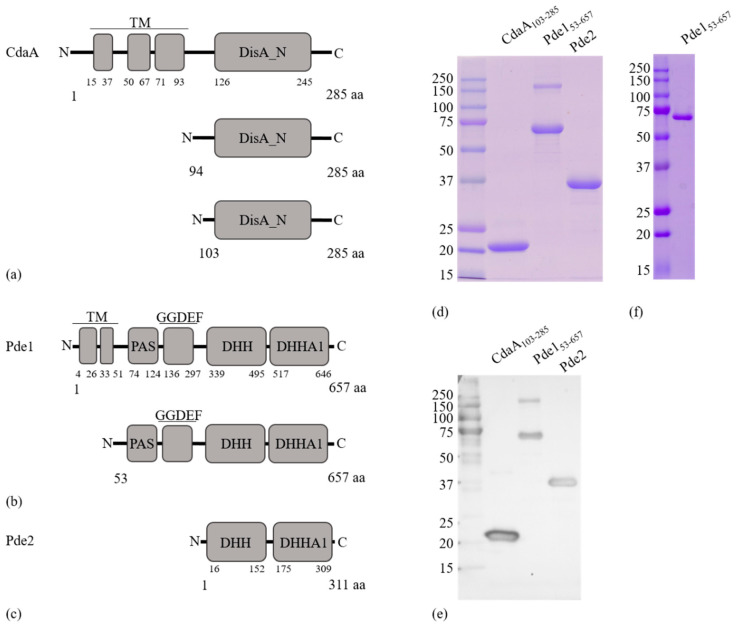
Schematic representation of protein domain organization and construct design. (**a**) Full-length CdaA protein and the expressed truncated recombinant proteins CdaA_94-285_ and CdaA_103–285_. (**b**) Full-length Pde1 and the expressed truncated version Pde1_53–657_. (**c**) The expressed full-length Pde2 protein. (**d**) Coomassie stained SDS-PAGE gel showing proteins purified by His-tag affinity followed by Size exclusion chromatography (SEC). (**e**) Western blot of proteins in (**d**) detected by a αHis-tag antibody. (**f**) Coomassie stained SDS-PAGE gel showing pure Pde1_53–657_ after purification using His-tag affinity. All expressed proteins had an N-terminal 6 x His-Tag included in their sequence which is not indicated in the figures. Legend: N, amino-terminus; C, carboxy-terminus; aa, amino acid; TM, Transmembrane region; DisA_N, Diadenylate cyclase domain; PAS, PAS domain; GGDEF, a degenerate diguanylate cyclase domain where GGDEF motif is changed to GGDQV; DHH, DHH domain; DHHA1, DHHA1 domain.

BLAST searches identified one homologue each of SPD_1392 (CdaA), SPD_2032 (Pde1) and SPD_1153 (Pde2) in *S. mitis* CCUG31611. We did not identify any homologues of the other classes of known diadenylate cyclases or phosphodiesterases. SM12261_1351 is predicted to consist of 285 amino acids and displayed 87.5% identity to SPD_1392 in the aligned part of the sequences (SPD_1392 is predicted to be only 271 aa [15]). SPD_2032 and SM12261_1779 are both predicted to be 657 aa and are 89% identical. SM12261_1122 is predicted to be 311 aa and 96% identical to SPD_1153. SM12261_1351, SM12261_1779, SM12261_1122 will hereafter be referred to as CdaA, Pde1, and Pde2, respectively. Prediction of the domain architecture revealed that CdaA contains three transmembrane α-helices in the N-terminus followed by a DisA_N domain (Figure 1a). Pde1 consisted of two transmembrane α-helixes, a PAS domain [38], a degenerate GGDEF domain [39], and a DHH/DHHA1 domain (Figure 1b). Pde2 contained only a DHH/DHHA1 domain (Figure 1c).

To assess the functions of CdaA, Pde1 and Pde2, N-terminally 6 x His-tagged versions of the recombinant proteins were expressed in *E. coli* BL21 (DE3). Since soluble full-length versions of CdaA, and Pde1 were produced at low levels, truncated versions of CdaA (CdaA_94–285_, CdaA_103–285_) and Pde1 (Pde1_53–657_) lacking the transmembrane helices together with a full length version of Pde2 were expressed, purified to homogeneity (Figure 1d–f) and used for functional assays. CdaA_94–285_ expressed at low levels relative to CdaA_103–285_ and was not used for further experiments. The molecular weights of the purified protein monomers corresponded with the expected theoretical values (CdaA_103–285_ = 21.21 kDa; Pde1_53–657_ = 68.19 kDa, Pde2 = 35.72 kDa) (Figure 1d). Moreover, size exclusion chromatography (SEC) demonstrated that CdaA_103–285_ and Pde2 migrate as single peaks with apparent MW of 42 kDa, and 92 kDa, respectively. Pde1_53–657_ on the other hand eluted in several peaks, starting at 168 kDa. These results indicate that CdaA_103–285_ and Pde2 dimerize in solution, whereas Pde1_53–657_ forms dimers as well as high-order oligomers. This correlates with previously published data for CdaA-, DhhP-, and GdpP-class enzymes [15,19,40,41].

**Figure 2:**
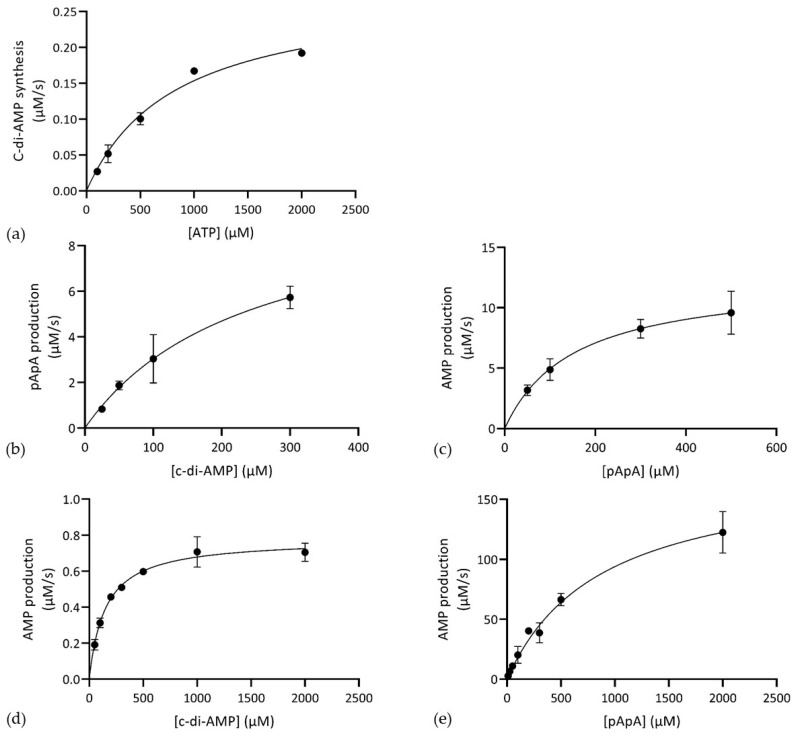
Steady-state kinetics of product formation by CdaA_103–285_, Pde1_53–657_ and Pde2. (**a**) C-di-AMP synthesis from ATP by CdaA_103–285_. (**b**) C-di-AMP hydrolysis to pApA by Pde1_53–657_. (**c**) pApA degradation to AMP by Pde1_53–657_. (**d**) C-di-AMP and (**e**) pApA hydrolysis to AMP by Pde2. Shown are average values and standard deviation of at least two individual experiments.

In vitro enzyme assays confirmed the predicted diadenylate cyclase activity of CdaA_103–285_ (Figure S1a). The reaction rate varied depending on the cofactors present (Mn^2+^ > Co^2+^ >> Mg^2+^). We performed kinetic analysis of CdaA_103–285_ in the presence of Mn^2+^ and it revealed that CdaA_103–285_ obeys Michaelis-Menten kinetics (Figure 2a and Table 2).

Interestingly, incubation of Pde1_53–657_ with c-di-AMP resulted in peaks corresponding to pApA as well as AMP (Figure S1b), demonstrating that Pde1 can hydrolyse c-di-AMP to AMP and indicating that pApA was an intermediate substrate in the hydrolysis of c-di-AMP. This was confirmed in an assay, using pApA as substrate (Figure S1c). Detailed enzyme kinetic analyses revealed that Pde1_53–657_ adhered to Michaelis-Menten kinetics for c-di-AMP conversion to pApA as well as for pApA hydrolysis to AMP and exhibited similar catalytic efficiency for both substrates (Figure 2b,c, Table 2).

Pde2 hydrolysed both c-di-AMP and pApA to AMP (Figures S1d and S2e) and pApA was not observed as an intermediate during c-di-AMP degradation (Figure S1d). Unlike Pde1_53–657_, the catalytic efficiency of Pde2 varied significantly with substrate and displayed much more efficient degradation of pApA compared to c-di-AMP (Figure 2d,e and Table 2).

### 3.2. Deletion Mutants Displayed Altered Intracellular Concentration of c-di-AMP and pApA

**Figure 3:**
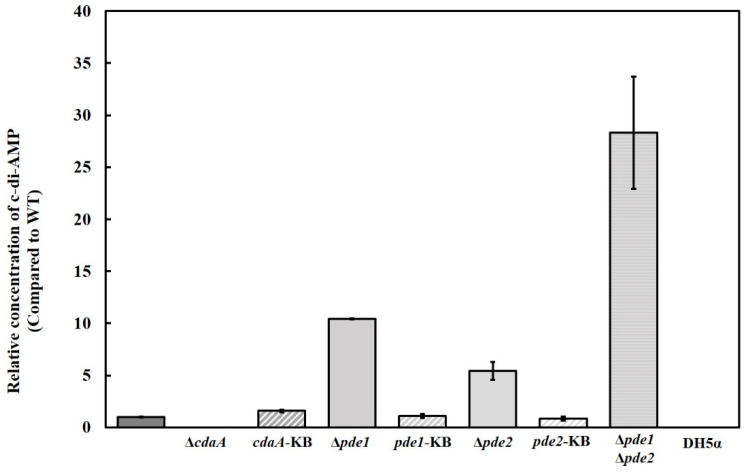
Intracellular concentration of c-di-AMP determined by HRMS-MS. Results are mean of at least three independent samples. Error bars represent standard error of the mean. *E. coli* DH5α was included as a negative control.

**Figure 4:**
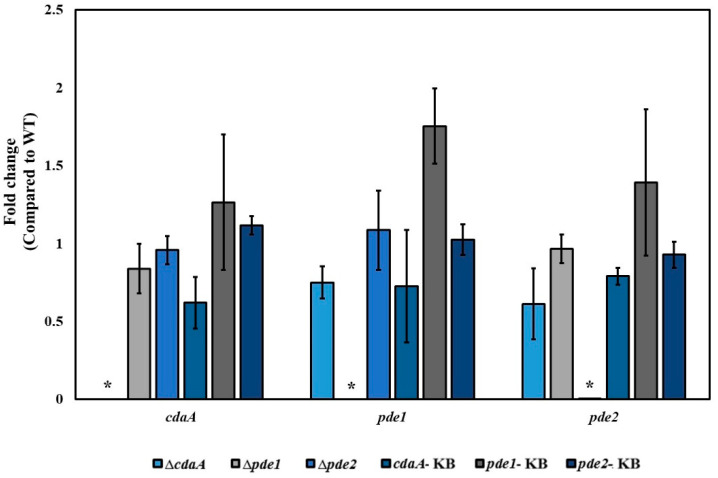
Transcription of genes related to c-di-AMP turn-over assessed by RT-qPCR. The gene encoding one of the subunits of DNA gyrase (*gyrA*) was used as reference gene. The relative transcription level of each gene in the mutants compared to the WT was calculated by the ΔΔCq-method. Shown are mean-values and standard error of the mean based on two independent experiments. * indicates statistical significance (*p* < 0.05).

The intracellular concentration of c-di-AMP was reduced in the Δ*cdaA*, whereas the Δ*pde1-* and Δ*pde2* mutants displayed a ten-fold and five-fold increase in c-di-AMP levels, respectively. Inactivation of both *pde1* and *pde2* resulted in an approximately 28-fold higher concentration of c-di-AMP in the Δ*pde1*Δ*pde2* double mutant compared to the WT (Figure 3). The intermediate metabolite of c-di-AMP hydrolysis, pApA was detected in the samples from the Δ*pde2* and Δ*pde1*Δ*pde2* mutants, but not in samples of the other strains. Since interference with the c-di-AMP signalling system has been shown to result in secondary mutations in many species [6,42,43,44], the gene of interest was reintroduced into the original locus on the chromosome to confirm that the phenotype was specific for the intended mutation. Successful re-introduction of the gene of interest was confirmed by PCR and Sanger sequencing. The resulting strains were referred to as knock back (KB) strains; *cdaA*-KB, *pde1*-KB and *pde2*-KB, respectively. Transcriptional analysis by RT-qPCR demonstrated that *cdaA*, *pde1* and *pde2* were transcribed in the respective KB-strains (Figure 4). Re-introduction of *cdaA*, *pde1* and *pde2* in the respective mutants restored the c-di-AMP concentration to WT-levels (Figure 3).

### 3.3. Deletion of pde2 Has a Pronounced Effect on S. mitis Colony Morphology

**Figure 5:**
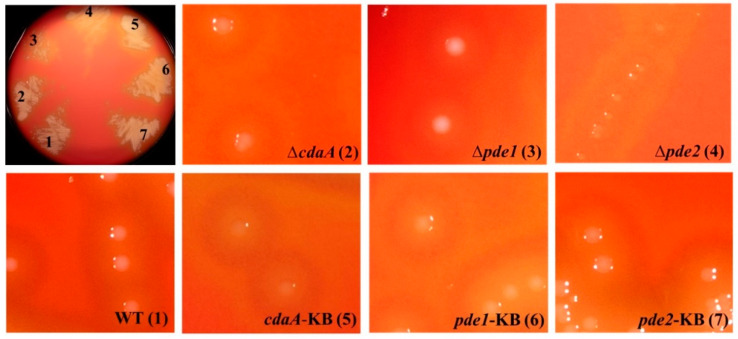
Disruption of the c-di-AMP signaling network affects colony morphology. Mutants and WT were grown on blood agar plates overnight in a humidified atmosphere containing 5% CO_2_. All mutants were grown on the same plate, and well-separated colonies were magnified. The brightness and contrast of the magnified pictures were manipulated to improve the contours of the colonies. The colors of the colonies and hemolysis are therefore not representative.

Deletion of *pde2* resulted in smaller colony size on blood agar plates, whereas colonies of the ∆*cdaA* and ∆*pde1* mutants were visually indistinguishable from the WT colonies (Figure 5). Re-introduction of *pde2* into the Δ*pde2* mutant (*pde2*-KB) restored normal colony morphology on blood agar similar to that of the WT (Figure 5).

### 3.4. The ∆pde2-Mutant Displayed Reduced Growth Rate

**Figure 6:**
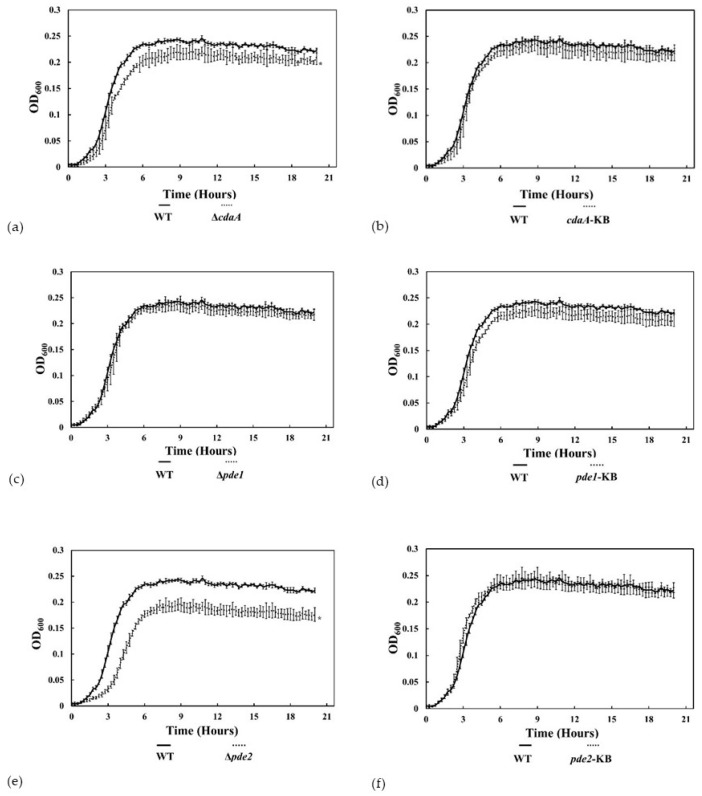
Disruption of the c-di-AMP signaling network affects growth in liquid culture. (**a**–**f**) Cultures were incubated in a plate reader and the optical density at 600 nm (OD_600_) was measured every 15 min for 20 h. Data are the mean of two independent experiments. * indicates statistically significant difference compared to WT. Error bars represent 95% confidence interval.

The smaller colony morphology on blood agar indicated that the growth of the Δ*pde2* mutant was reduced compared to the WT. To analyse this growth defect in more detail, bacteria were inoculated in TSB and incubated at 37 °C for 20 h. There was a significant difference in growth between the WT and the Δ*cdaA* mutant in late exponential phase and the Δ*cdaA* mutant reached a significantly lower OD in stationary phase (Figure 6a). Growth of the Δ*cdaA* mutant was restored in the *cdaA*-KB (Figure 6b). There was no difference in growth between the Δ*pde1* mutant, the *pde1*-KB strain and the WT (Figure 6c,d). The ∆*pde2*-mutant grew significantly slower in exponential phase compared to the WT and reached a lower OD in stationary phase (Figure 6e). Growth was restored to WT-levels in the *pde2*-KB strain (Figure 6f). Calculations of the doubling time in exponential growth phase revealed that the ∆*pde2*-mutant had a generation time of 54.8 min, approximately 1.4 times longer than the WT (39.6 min) (Table 3).

The ∆*pde1*-mutant also displayed a longer generation time (42 min) than the WT, and this was compensated in *pde1*-KB (39.4 min). The generation times of ∆*cdaA* and *cdaA*-KB were almost indistinguishable from the WT, with 38.7 min and 37.3 min, respectively. The *pde2*-KB had the fastest doubling time (33.8 min).

### 3.5. The ∆pde2 Mutant Displayed Reduced Rate of Glucose Metabolism

**Figure 7:**
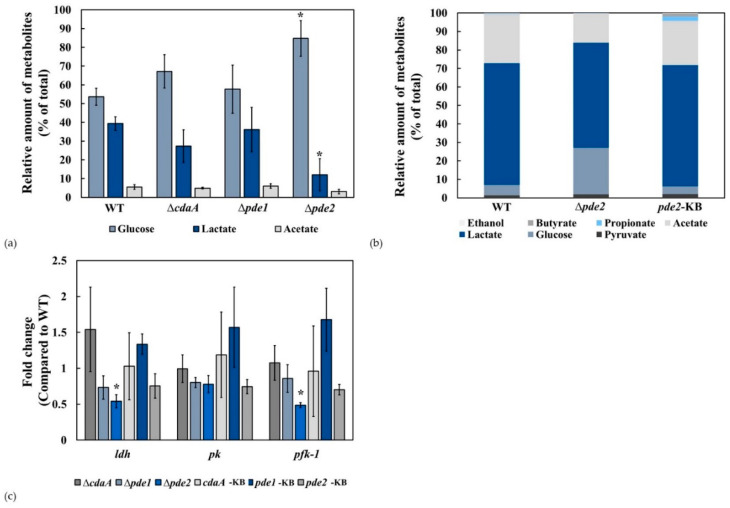
Metabolism of glucose is slower in the ∆*pde2*-mutant. (**a**) The relative concentration of ^14^C-labelled glucose and the major metabolites lactate and acetate was determined for the WT, Δ*cdaA*-, Δ*pde1*-, and Δ*pde2*-mutants. * Students t-test *p* < 0.05. (**b**) The reduced glucose metabolism of the Δ*pde2* mutant was restored in the *pde2*-KB strain. The levels of glucose and lactate was significantly different (*p* < 0.05) in the Δ*pde2*-mutant compared to WT (**c**) Transcription of genes related to glucose metabolism were investigated by RT-qPCR. Total RNA was extracted from liquid cultures, cDNA was synthesized and qPCR was performed with primers for phosphofructokinase-1(*pfk-1*), lactate dehydrogenase (*ldh*), pyruvate kinase (*pk*) and *gyrA* as the reference gene. Data are represented as the fold change in the Δ*cdaA*- Δ*pde1*-, Δ*pde2* mutants and the *cdaA*-KB, *pde1*-KB and *pde2*-KB strains compared to the WT calculated by the ΔΔCq-method. Shown are means and standard errors of the mean from two independent experiments. * indicates statistical significance (*p* < 0.05).

We hypothesised that the growth defect in the Δ*pde2* mutant was due to reduced metabolic activity and decided to analyse the rate of glucose metabolism in exponentially growing bacteria. Glucose metabolism was reduced in the ∆*pde2*-mutant compared to the WT, Δ*cdaA* and Δ*pde1* mutants (Figure 7a). Knock back of the *pde2*-gene (*pde2*-KB) restored glucose metabolism to the WT level (Figure 7b).

The results of the metabolism experiments indicated that one or more steps of glycolysis were affected. We investigated the transcriptional activity of genes encoding enzymes catalysing rate limiting steps in glycolysis. Transcription of phosphofructokinase-1 (*pfk-1*) and lactate dehydrogenase (*ldh*) was reduced in the Δ*pde2* mutant compared to WT and restored to WT-levels in the *pde2*-KB strain (Figure 7c). The transcription level of the gene encoding pyruvate kinase (*pk*) was similar in all tested strains.

### 3.6. Interference with c-di-AMP Signaling Affects Chain Length

**Figure 8:**
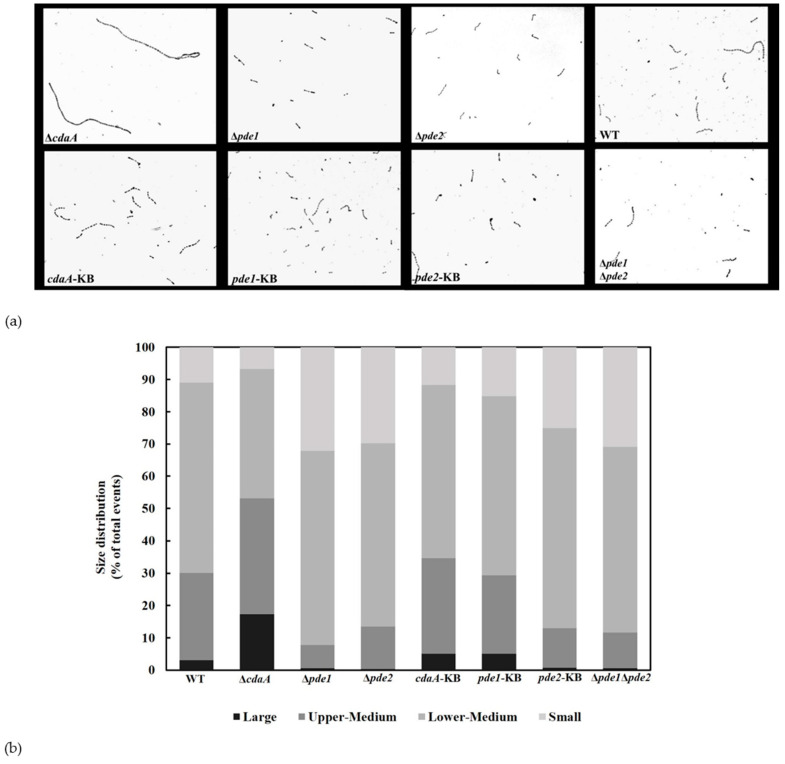
C-di-AMP is involved in regulating chain length. (**a**) Micrographs of safranin-stained cultures grown to OD_600_ of 0.5 and photographed with a light microscope (Nikon). (**b**) Flow cytometry was used to sort cells in the culture based on forward scatter and side scatter. Results were gated into four populations, denoted small, lower-medium, upper-medium and large. Results show the average size distribution of the samples in two independent experiments.

Streptococci generally grow in pairs or chains. Bai et al. have previously reported that an increase in intracellular c-di-AMP concentration resulted in slightly shorter chain-lengths in *S. pneumoniae* [15]. Based on this, we investigated the chain length of the *S. mitis* WT, mutants and KB-strains in mid-exponential phase. Representative micrographs are shown in Figure 8a. Although all strains displayed diverse chain lengths, there was a visible difference in chain length between WT and Δ*cdaA.* In the Δ*pde1*, Δ*pde2* and Δ*pde1*Δ*pde2* mutants the chain length appeared to be shorter compared to the WT with few long chains.

To complement the visual analysis of chain length with an automated objective method, flow cytometry was performed on bacteria growing in mid-exponential phase. The bacteria were analyzed based on their size and granularity (forward and side scatter) (Figure S2a–h). This allowed us to sort the counted events into four sub-populations based on size (small, mid-small, mid-large, and large). The flow cytometry analysis showed that the ∆*cdaA* mutant had a higher proportion of events in the larger populations compared to WT (Figure 8b). The ∆*pde1*, ∆*pde2 and* ∆*pde1*∆*pde2* mutants had a higher distribution of short and lower-medium sized events compared to the WT (Figure 8b).

### 3.7. Transformation Efficiency Was not Affected by Disruption of the c-di-AMP Signalling System

*S. mitis* is naturally genetically competent which means that it can take up and integrate foreign DNA into its genome. The transformation efficiency was measured by the ability to acquire erythromycin resistance from free DNA in the environment of the bacteria. There was a trend towards increased transformation efficiency in the ∆*cdaA* mutant compared to the WT and decreased transformation efficiency in the ∆*pde1* and the ∆*pde2* mutants. However, the differences were relatively small and not statistically significant.

## 4. Discussion

In this study, we identified one CdaA-type diadenylate cyclase, one GdpP-type phosphodiesterase (Pde1) and one DhhP-type phosphodiesterase (Pde2) involved in regulating the c-di-AMP concentration of *S. mitis* CCUG 31611. This set of proteins involved in c-di-AMP turnover is preserved in all streptococci in which c-di-AMP signalling has been studied to date. We created single markerless in-frame deletion mutants of each of *cdaA*, *pde1* and *pde2* by a standard protocol that utilizes the natural competence of *S. mitis* [33]. Our success in creating a *cdaA*-deletion mutant was somewhat unexpected, since it has proven difficult to inactivate the sole diadenylate cyclase CdaA in several streptococci by conventional protocols. Whereas deletion of *cdaA* was possible in *S. pyogenes* [3] and *S. mutans* [45,46], all attempts to delete *cdaA* have been unsuccessful in *S. pneumoniae* [15] and *S. gallolyticus* [47]. In *S. agalactiae*, it was only possible to delete the chromosomal copy of *cdaA* in a strain carrying an inducible copy of *cdaA* on a plasmid [44]. This suggests that c-di-AMP is essential in certain species of streptococci and under certain conditions but dispensable in other species, including *S. mitis*. The reason for this discrepancy between species of streptococci is not known.

In our in vitro enzymatic analyses, CdaA_103–285_ displayed diadenylate cyclase activity, and adhere to Michaelis-Menten kinetics. CdaA_103–285_ was active in the presence of Mn^2+^ or Co^2+^ but displayed very low activity with Mg^2+^ as cofactor, which correlates with previous reports [15,48,49]. Interestingly, both Pde1_53–657_ and Pde2 were capable of hydrolysing c-di-AMP to pApA and further to AMP. It has previously been shown that GdpP-type phosphodiesterases degrade c-di-AMP exclusively to pApA [4,15,17,24,50,51]. However, based on structural comparison Wang et al. engineered GdpP-type and DhhP-type PDEs with altered substrate preferences and elegantly demonstrated that a single amino acid alteration in the nucleotide binding site of GdpP made hydrolysis of pApA to AMP possible [52]. Thus to our knowledge, Pde1 of *S. mitis* is the only GdpP-type phosphodiesterase that in its original structure has been demonstrated to degrade c-di-AMP to pApA and further to AMP in vitro. We did not detect pApA during c-di-AMP degradation by Pde2, which correlated with a higher catalytic efficiency for pApA compared to c-di-AMP. This is in general agreement with previous publications [15,23,40,53,54,55,56,57,58].

Quantification of the intracellular concentration of c-di-AMP revealed that deletion of *cdaA* resulted in reduced levels of c-di-AMP, whereas deletion of *pde1* and *pde2* increased the c-di-AMP concentration. The c-di-AMP concentration of the Δ*pde1*Δ*pde2* mutant was further elevated compared to the Δ*pde1* and Δ*pde2* mutants, which demonstrates that both phosphodiesterases perform a functional role in vivo under the conditions tested. We also detected pApA, the intermediate product of c-di-AMP metabolism in the Δ*pde2* mutant but not in the Δ*pde1* mutant, which is consistent with the high catalytic efficiency of Pde2 in degradation of pApA demonstrated in vitro. This indicates that although Pde1_53–657_ can degrade both c-di-AMP and pApA at similar efficiency in vitro Pde1 preferentially degrades c-di-AMP in vivo. Unexpectedly, we also detected pApA in the Δ*pde1*Δ*pde2* mutant. This may indicate that *S. mitis* CCUG31611 encodes a novel class of phosphodiesterases. Similar results were observed in a Δ*gdpP*Δ*pde2* mutant of *S. aureus* and the authors speculated that another phosphodiesterase was responsible for production of pApA, or that pApA was generated in a c-di-AMP independent pathway [58].

We utilized the aforementioned markerless in-frame deletion mutants of each of the genes encoding c-di-AMP turnover proteins to study the physiological role of c-di-AMP in *S. mitis*. The deletion mutant of *pde2* grew in smaller colonies on blood agar compared to the WT, whereas the Δ*cdaA* and Δ*pde1* mutants displayed a colony morphology similar to the WT. Since the WT-colony morphology was restored in the *pde2*-KB strain, the altered colony morphology of the Δ*pde2* mutant was not due to secondary mutations. A smaller colony phenotype was also reported in *Mycobaterium smegmatis* when the diadenylate cyclase DisA was overexpressed [57]. Contrary to our observations, the small colony morphology was only present in the first generation and was restored to the WT phenotype following continuous culture. In *B. anthracis* a small colony phenotype was observed when both of its phosphodiesterases were deleted [14]. Taken together, these results indicate that increased c-di-AMP concentrations results in growth defects on agar.

We decided to study the c-di-AMP mediated effects on growth in more detail in broth cultures. Deletion of *pde1* did not affect growth significantly, while deletion of *cdaA* resulted in significantly lower cell density in stationary phase. Deletion of *pde2* resulted in considerably slower growth of *S. mitis*, which is in accordance with observations made for the Δ*pde2* mutant of *S. pneumoniae* [15]. Considering that the Δ*pde1* mutant displayed higher c-di-AMP concentration than the Δ*pde2* mutant, it is possible that the growth defect of the Δ*pde2* mutant is a consequence of high intracellular concentration of pApA, but this requires further investigation. Growth was restored in the *pde2*-KB strain, demonstrating that the growth defect was specifically caused by the *pde2* mutation. Since streptococci encompass a fragmentary TCA cycle, they preferentially extract energy from fermentation of glucose to organic acids even in aerobic conditions. The Δ*pde2* mutant displayed reduced glycolytic activity compared to the WT. In an attempt to determine which step of glycolysis was affected, we performed RT-qPCR of selected genes encoding key enzymes of the pathway and detected a small reduction in transcription of *pfk-1* and *ldh* but not *pk* in the Δ*pde2* mutant. Considering that, the relative distribution of the major metabolites lactate and acetate was not affected to a large extent we hypothesise that a rate-limiting step of glycolysis upstream of pyruvate was inhibited, which correlates with reduced expression of *pfk-1*. However, we cannot exclude that the reduction in glucose metabolism was due to reduced uptake of glucose or direct post-translational inhibition of a glycolytic enzyme. It has for example been shown that pyruvate carboxylase (PC) that catalyzes the anapleurotic reaction transforming pyruvate to oxaloacetate is allosterically inhibited by c-di-AMP in *L. monocytogenes* [59], but *S. mitis* does not encode PC.

*S. mitis* generally grow in chains under standard laboratory conditions. Investigation of chain length revealed differences between the numbers of cells in the chains of the Δ*cdaA*, Δ*pde1* and Δ*pde2* mutants compared to WT. We observed that Δ*cdaA* grew in significantly longer chains, while Δ*pde1* and Δ*pde2* grew in shorter chains. Since the chain lengths were restored to WT-level in the *cdaA*-KB, *pde1*-KB and *pde2*-KB these effects were specific for the respective gene deletions. In line with our observations, deletion of *pde1* and *pde2* in *S. pneumoniae* D39 resulted in shorter chains compared to the WT and deletion of the gene encoding the Pde2 ortholog PapP shortened the long chains of *S. pneumoniae* TIGR4, to diplococci [15,56]. Kuipers et al. showed that this correlated with mislocalization of the important cell-division proteins FtsA and FtsZ in the *papP* deletion mutant, and concluded that this was due to changes in membrane composition as a consequence of altered fatty acid synthesis [56].

## Figures and Tables

**Table 1:** Strains, mutants and plasmids used in this study.

Name	Description	Origin	Reference
*S. mitis* CCUG 31611	Type strain *Streptococcus mitis*, corresponding to NCTC 12261 and ATCC 49456		CCUG
MI009	∆*srtA*::erm; Erm^R,^. Used to prepare an amplicon of the erythromycin resistance cassette and flanking regions used in transformation efficiency assays	*S. mitis* CCUG 31611	[33]
*S. mitis* Δ*cdaA*	Markerless in-frame deletion of SM12261_1351	*S. mitis* CCUG 31611	This study
*S. mitis* Δ*pde1*	Markerless in-frame deletion of SM12261_1779	*S. mitis* CCUG 31611	This study
*S. mitis* Δ*pde2*	Markerless in-frame deletion of SM12261_1122	*S. mitis* CCUG 31611	This study
*S. mitis* Δ*pde1*Δ*pde2*	Markerless in-frame deletion of SM12261_1779 and SM12261_1122	*S. mitis* Δ*pde2*	This study
*S. mitis cdaA*-KB	SM12261_1351 re-introduced into the original locus	*S. mitis* Δ*cdaA*	This study
*S. mitis pde1*-KB	SM12261_1779 reintroduced into the original locus	*S. mitis* Δ*pde1*	This study
*S. mitis pde2*-KB	SM12261_1122 re-introduced into the original locus	*S. mitis* Δ*pde2*	This study
*E. coli* DH5α	Strain used for cloning	-	Thermo Fisher Scientific
*E. coli* BL21 (DE3)	Strain used for expression of recombinant proteins.	-	Thermo Fisher Scientific
pET28a(+)	Expression vector with IPTG-inducible T7-promoter; Kan^R^		Novagen
pET28a(+)KpnI-mut	pET28a(+)-vector with the NcoI restriction site mutated to KpnI; Kan^R^	pET28a(+)	This study
pET28a(+)KpnI-mut_CdaA-N-His	pET28a(+)KpnI-mut-vector carrying the full-length *S. mitis cdaA* ORF; Kan^R^	pET28(+)KpnI-mut	This study
pET28a(+)KpnI-mut_CdaA-T-N-His	pET28a(+)KpnI-mut-vector carrying the *S. mitis cdaA* including aa 94-285 with an N-terminal 6x His-tag; Kan^R^	pET28(+)KpnI-mut	This study
pET28a(+)KpnI-mut_CdaA-T2-N-His	pET28a(+)KpnI-mut-vector carrying the *S. mitis cdaA* including aa 103–285 with an N-terminal 6x His-tag; Kan^R^	pET28(+)KpnI-mut	This study
pET28a(+)KpnI-mut_Pde1-N-His	pET28a(+)KpnI-mut-vector carrying the full-length *S. mitis pde1* with an N-terminal 6x His-tag; Kan^R^	pET28(+)KpnI-mut	This study
pET28a(+)KpnI-mut_Pde1-T-N-His	pET28a(+)KpnI-mut-vector carrying the *S. mitis pde1* ORF including aa 53–657 with an N-terminal 6x His-tag; Kan^R^	pET28a(+)KpnI-mut_PDE1-N-His	This study
pET28a(+)KpnI-mut_Pde2-N-His	pET28a(+)KpnI-mut-vector carrying the full-length *S. mitis pde2* with an N-terminal 6x His-tag; Kan^R^	pET28(+)KpnI-mut	This study

**Table 2:** Enzyme kinetics parameters/results.

Enzyme	Substrate	Product	[Enzyme] (μM)	Km (μM)	Vmax (μM s^−1^)	Kcat (s^−1^)	kcat/Km (μM^−1^ s^−1^)
**CdaA_103–285_**	ATP	c-di-AMP	10	821.3 ± 10.5	0.280 ± 0.046	28 × 10^−3^ ± 4.6 × 10^−3^	3.41 × 10^−5^
**Pde1_53–657_**	c-di-AMP	pApA	2.5	241.3 ± 11.6	10.35 ± 4.98	4.14 ± 1.99	17 × 10^−3^
	pApA	AMP	5	152.4 ± 03.6	12.47 ± 3.06	2.49 ± 0.61	16.4 × 10^−3^
**Pde2**	c-di-AMP	AMP	10	148.5 ± 34.8	0.78 ± 0.05	0.078 ± 0.005	5.25 × 10^−4^
	pApA	AMP	0.05	877 ± 312	176.4 ± 31.2	3527 ± 623	4.02

**Table 3:** Generation time.

Strain	Generation Time (min) (Standard Error of the Mean)
WT	39.6 ± 0.5
∆*cdaA*	38.7 ± 2.3
*cdaA*-KB	37.3 ± 1.0
∆*pde1*	42.0 ± 0
*pde1*-KB	39.4 ± 1.1
∆*pde2*	54.8 ± 0.6
*pde2*-kb	34.2 ± 0.4

Generation time was calculated from the exponential phase of the independent growth curves combined and depicted in Figure 6.

## Supplementary Materials

The following are available online at https://www.mdpi.com/2076-2607/8/9/1269/s1, Figure S1: Enzymatic activities of *S. mitis* CdaA_103–285_, Pde1_53–667_ and Pde2. Figure S2: Flow cytometry was used to compare size and granularity of WT and mutants. Table S1: Primers used in this study.
