# Supplementary material for: Cyclic Di-adenosine Monophosphate Regulates Metabolism and Growth in the Oral Commensal Streptococcus mitis
Source: Microorganisms. 2020 Aug 20;8(9):1269. doi: 10.3390/microorganisms8091269 (PMC7570391; doi:10.3390/microorganisms8091269)
Supplement: Supplementary file 1 [file microorganisms-08-01269-s001.pdf]

Supplementary Materials

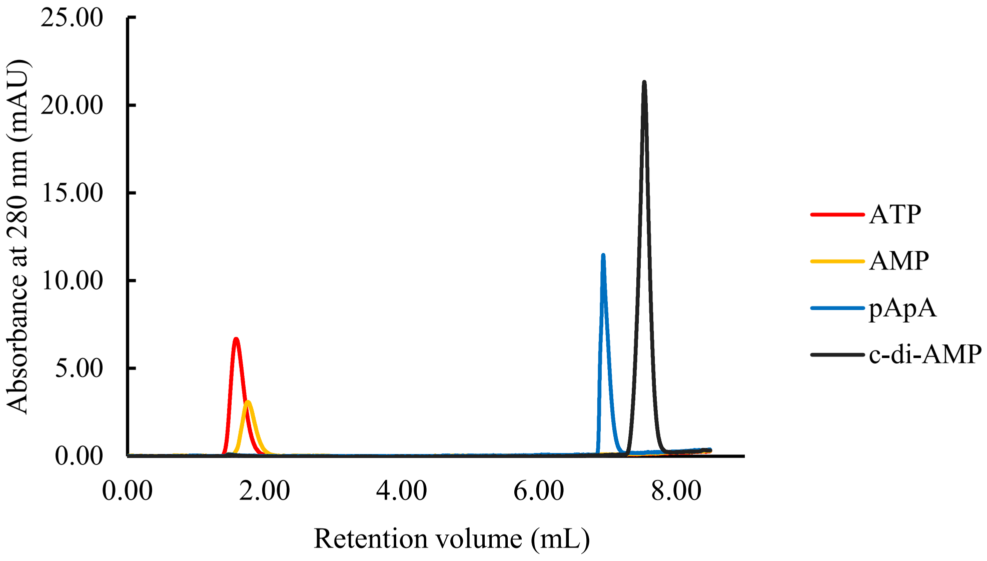

(a)

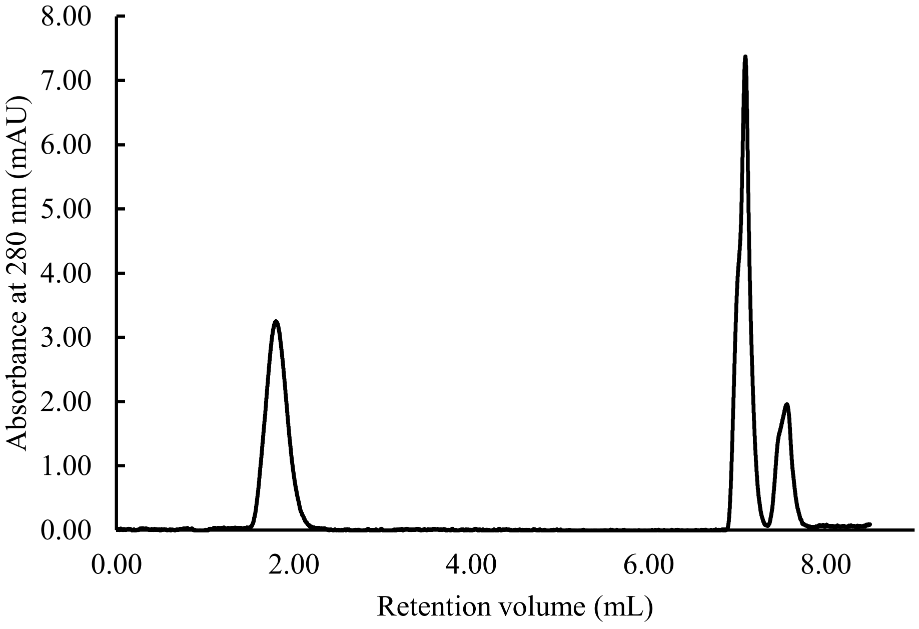

(b)

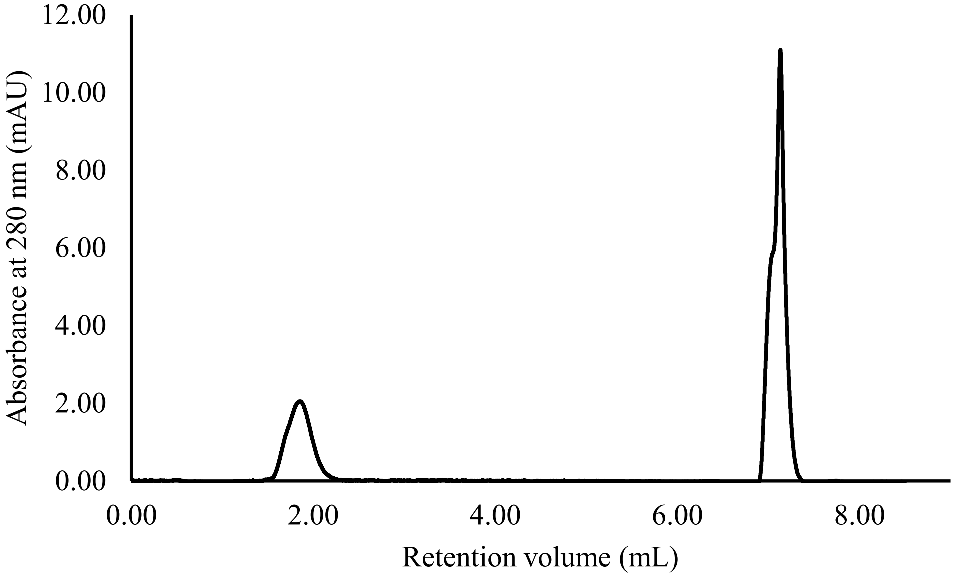
12.00
10.00
8.00
6.00
4.00
2.00
0.00
Absorbance at 280 nm (mAU)
0.00
2.00
4.00
6.00
8.00
Retention volume (mL)

(c)

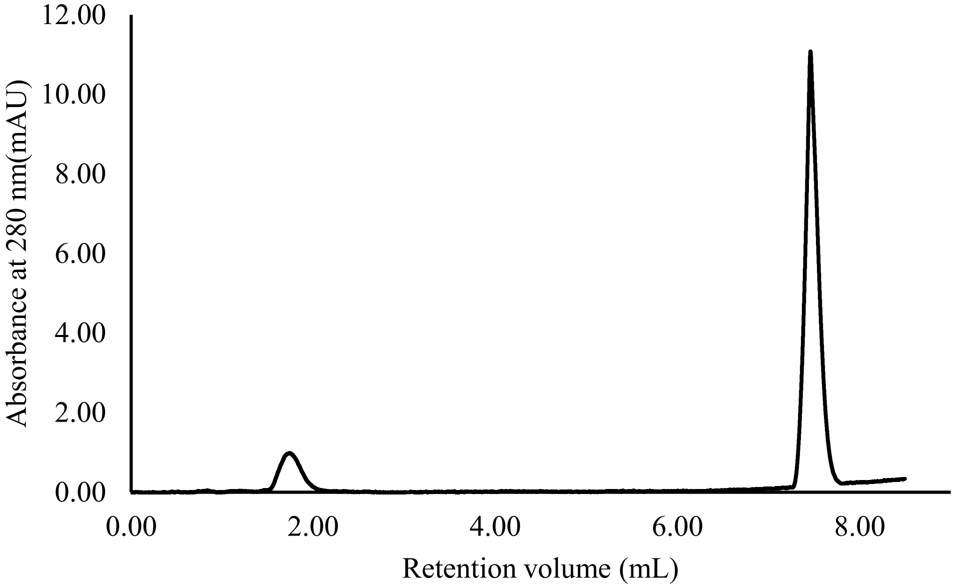
12.00
10.00
8.00
6.00
4.00
2.00
0.00
Absorbance at 280 nm(mAU)
0.00
2.00
4.00
6.00
8.00
Retention volume (mL)

(d)

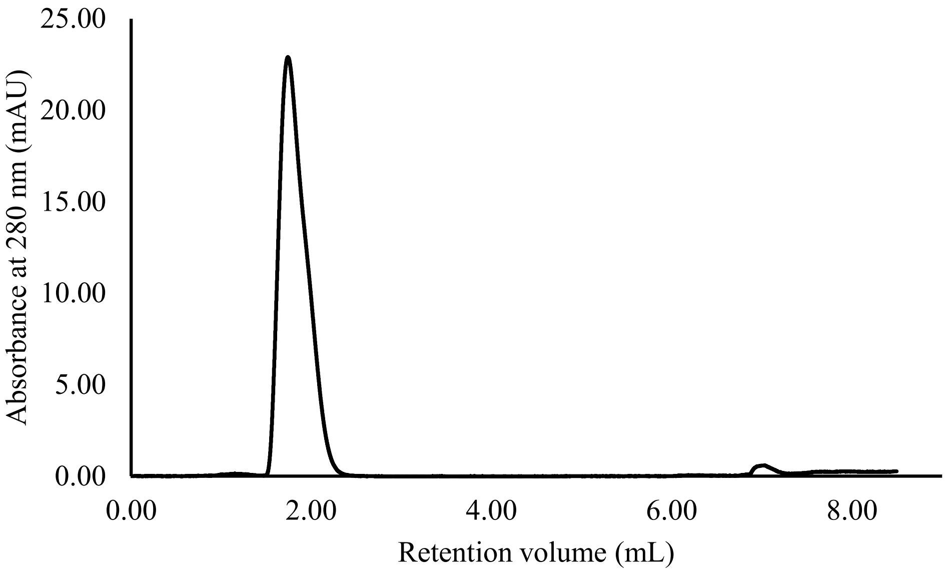

(e)

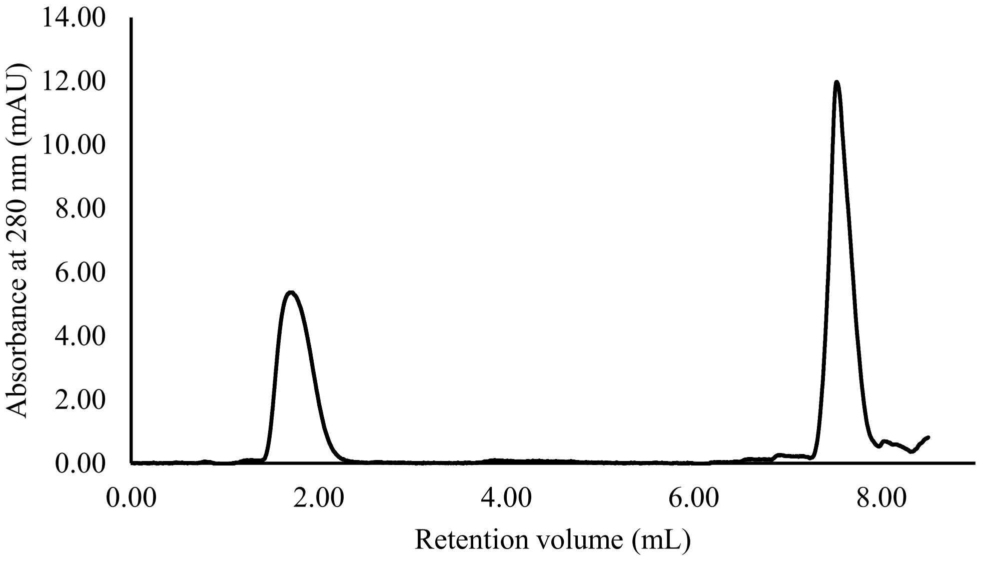

(f)

Figure S1. Enzymatic activities of *S. mitis* CdaA$_{103-285}$, Pde1$_{53-667}$ and Pde2. (a) Nucleotide control peaks for ATP, AMP, pApA and c-di-AMP eluted separately with the reversed-phase chromatography method. The x-axis of the chromatogram shows the retention volume in milliliters (mL) and y-axis shows the absorbance at 280 nm in milli-absorbance units (mAU). (b). Pde1$_{53-657}$ degrading c-di-AMP to pApA and AMP. (c). Pde1$_{53-657}$ degrading pApA into AMP. (d). Pde2 degrading c-di-AMP directly into AMP (e), and pApA into AMP (f). CdaA$_{103-285}$ synthesis of c-di-AMP from ATP.

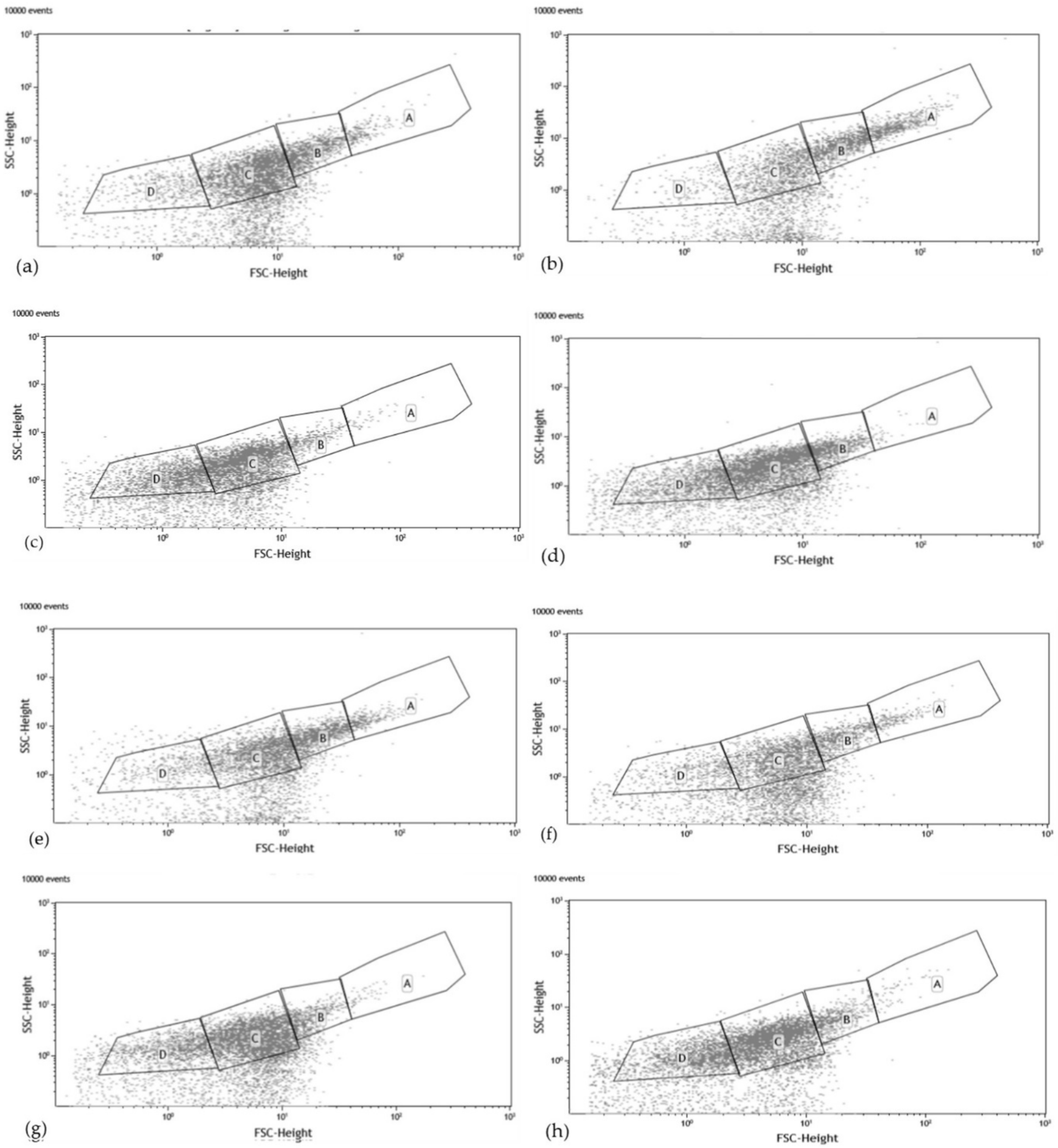

Figure S2. Flow cytometry was used to compare size and granularity of WT and mutants. Scatter plots from one representative experiment. (a) WT. (b) Δ*cdaA.* (c) Δ*pde1.* (d) Δ*pde2.* (e) *cdaA*-KB. (f) *pde1*-KB. (g) *pde2*-KB. (h) Δ*pde1*Δ*pde2.*

Table S1. Primers used in this study.

Primer	Sequence (5′- 3′)	Description
**Gene deletion**		

cdaA-KO-L for<br>cdaA-KO--L-rev	AGGATGGGACAGAAGTGGC<br>TATTTTTTCTCATCTGCTGTCTCCTCTCTGTC	Amplification of the upstream-flanking DNA region of *cdaA*
cdaA-KO-R-for<br>cdaA-KO-R-rev	GACAGCAGATGAGAAAAAATAGTCTATATATC<br>TCTCGCCAGTTAAAGACAC	Amplification of the downstream-flanking DNA region of *cdaA*
PDE1-KO-L for<br>PDE1-KO-L-rev	CCACTGGGCTCACCATC<br>AAAGATTACTTTCCAAACCTCTTGGCACC	Amplification of the upstream-flanking DNA region of *pde1*
PDE1-KO-R-for<br>PDE1-KO-R-rev	GAGGTTTGGAAAGTAATCTTTTTAGCAGATGT<br>CCATAGTTAGAACTATTCCC	Amplification of the downstream-flanking DNA region of *pde1*
PDE2-KO-L for<br>PDE2-KO-L-rev	CTCACTTACCTCACCAAAG<br>CAAGTATTTTAAAATAACCTCAATTCTTTCTCA	Amplification of the upstream-flanking DNA region of *pde2*
PDE2-KO-R-for<br>PDE2-KO-R-rev	TTGAGGTTATTTTAAAATACTTGCCAAACTTTTC<br>TGTGCTTGGCGCATCATG	Amplification of the downstream-flanking DNA region of *pde2*
GHR17	ACAGGACCACCACCTACAAT	Screen for *cdaA* knock out - Forward
GHR18	CAAGGTCACACGATTAGCTC	Screen for *cdaA* knock out - Reverse
GHR19	CCAATGTGATTTATCGTCGT	Screen for *pde1* knock out - Forward
GHR20	TTAATTGCTTTTGCTTCTGC	Screen for *pde1* knock out - Reverse

GHR21	ATCAACATCAACATCCAAGC	Screen for *pde2* knock out - Forward
GHR22	ACGGATCAATGGGTAAGTTT	Screen for *pde2* knock out - Reverse
FP1242	CTTGAGCTGGGCTTCGTAGT	Amplification of Erythromycin cassette Forward
FP1243	ACAGGGGATGTCATGGGTAA	Amplification of Erythromycin cassette Reverse
**RT-qPCR**		
GHR27	AAGGGCGTGACTAAGGTCAA	Amplification of *ldh*
GHR28	TATCCTTGTCGGTGATGGTG	
GHR29	CGTTGCAACTTTGGGACCTG	Amplification of *pk*
GHR30	AGTTGAAACGGAATGTGTTAGCA	
GHR31	GAGACGCCCCTGGTATGAAC	Amplification of pfk-1
GHR32	GTCCCCTACTGAAGCTGCAT	
GHR1	GGGCCAGAGTCATCTGGTAA	Amplification of *recA*
GHR2	CGTCAATGTTGACACCAAGG	
GHR3	GATGGTCATGGGAACTTTGG	Amplification of *gyrA*
GHR4	TTCACGTTCATTGGCATCAT	
GHR5	GAAAGCACAGGGATTTCCAA	Amplification of *cdaA*
GHR6	CGTGCTTGAAAACACCGTTA	
GHR7	GAACAGATGCCTGTCGGAGT	Amplification of *pde1*

GHR8	GATTCCCCACAGAAGCCTTT	
GHR9	CTTGGATGGCTGAGATGGAT	Amplification of *pde2*
GHR10	CATCATTTGGATGGTGGTCA	
**Cloning**		
pET28a(+)-Seq-F	CCGGCCACGATGCGTCC	pET28a(+) sequencing primers
pET28a(+)-Seq-R	ACAGGGCGCGTCCCATTC	
pET28a(+)-KpnI-mut-F	AGAAGGAGAGGTACCATGGGCAGCAGCCATCATCATCATCATCAC	Site-directed mutagenesis of a restriction site from NcoI to KpnI
pET28a(+)-KpnI-mut-R	CCCATGGTACCTCTCCTTCTTAAAGTTAAACAAAATTATTTCTAGAGGGGAATTGTT	
DisA-HindIII-R	TATCAAGCTTCTATTTTTTCTCATATTTCCAAC	Full-length *cdaA*
DisA-N-His-F	GATCGGTACCATGCATCACCATCACCATCACAACTTTCAACAATTATCC	Full-length *cdaA* with an N-terminal 6x His-Tag
DisA-T-N-His-F	GATCGGTACCATGCATCACCATCACCATCACCCAGAGATTCGGACTG	*cdaA*$_{94\text{-}285}$ with an N-terminal 6x His-Tag
PDE1-HindIII-R	TATCAAGCTTCTATCATTCTTCTTTCTCCTTTTCCT	Amplification of full-length and truncated *pde1*
PDE1-N-His-F	GATCGGTACCATGCATCACCATCACCATCACAAAAAATTTTATGTAAGTCCTATTTTC	Full-length *pde1* PCR and addition of an N-terminal 6x His-Tag – F

PDE1-midseq-F	TTCGTAGTGTGGATCAGGTT	*pde1* sequencing primer
PDE1_Truncated-N-His-F	GATCGGTACCATGCATCACCATCACCATCACCAGAGAGACTACTACTCAAAG	*pde1*$_{53\text{-}657}$ with an N-terminal 6x His-Tag - F
PDE2-HindIII-R	TATCAAGCTTCTATCAGTTTTTAAGCAAGTTTTTTAAC	Full-length *pde2* PCR
PDE2-N-His-F	GATCGGTACCATGCATCACCATCACCATCACGACATTTGCCATCAAATTTTAG	Full-length *pde2* with an N-terminal 6x His-Tag - F
SMITIS_CdaA2-For	CCATCACCATTTGGGAAGGGCGACAGATTTCTTTTCTACTGCTCAAATTAG	Site-directed deletion of *cdaA* aa residues 94-102
SMITIS_CdaA2-Rev	CCCTTCCCAAATGGTGATGGTGATGGTGCATGGTATATCTCCTTCTTAAAG	Site-directed deletion of *cdaA* aa residues 94-102
